# Supplementary material for: Vibrational Study on the Structure, Bioactivity, and Silver Adsorption of Silk Fibroin Fibers Grafted with Methacrylonitrile
Source: Molecules. 2023 Mar 10;28(6):2551. doi: 10.3390/molecules28062551 (PMC10051891; doi:10.3390/molecules28062551)
Supplement: Supplementary file 1 [file molecules-28-02551-s001.zip › molecules-2254787-supplementary REVISED.pdf]

Table S1. Weight gain % of the silk fibers under study after ageing in the Ag^{+}-containing solution for 48h.

Sample	Weight gain %, t = 48h
control	2.81
MAN 10%	0.01
MAN 25%	0.66
MAN 32.5%	1.06
MAN 40%	1.65
MAN 60%	3.82

Table S2. Weight gain % of the silk fibers under study after 7 and 28 days in the SBF solution.

Sample	Weight gain %, t = 7 days	Weight gain %, t = 28 days
control	-1.53	-5.95
MAN 10%	-1.49	
MAN 25%	-0.97	
MAN 32.5%	0.16	
MAN 40%	0.94	-4.12
MAN 60%	1.08	1.66

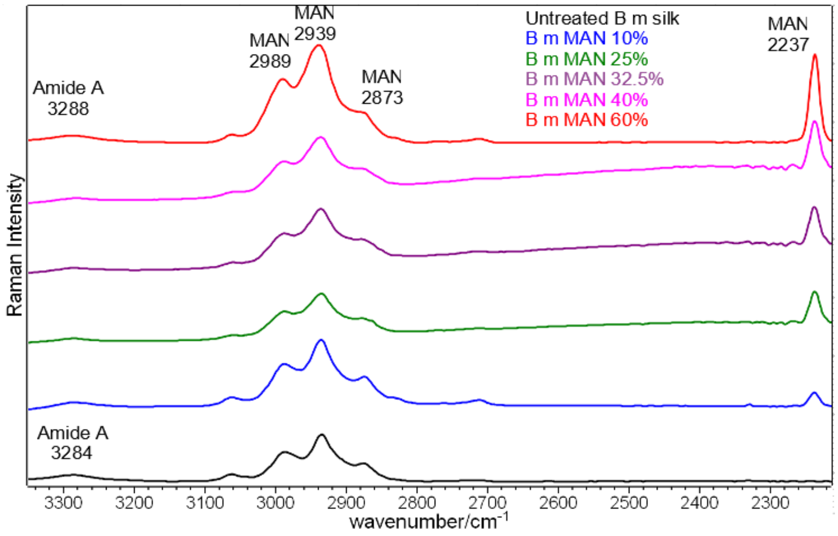

(**a**)

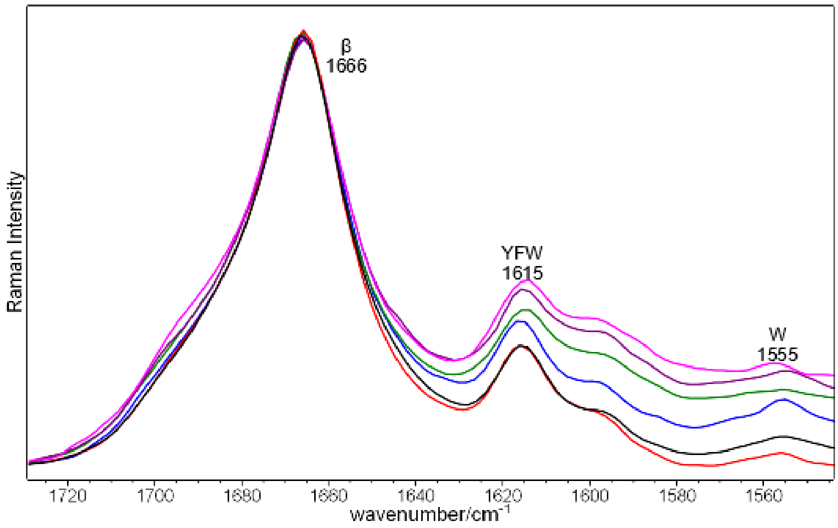

(**b**)

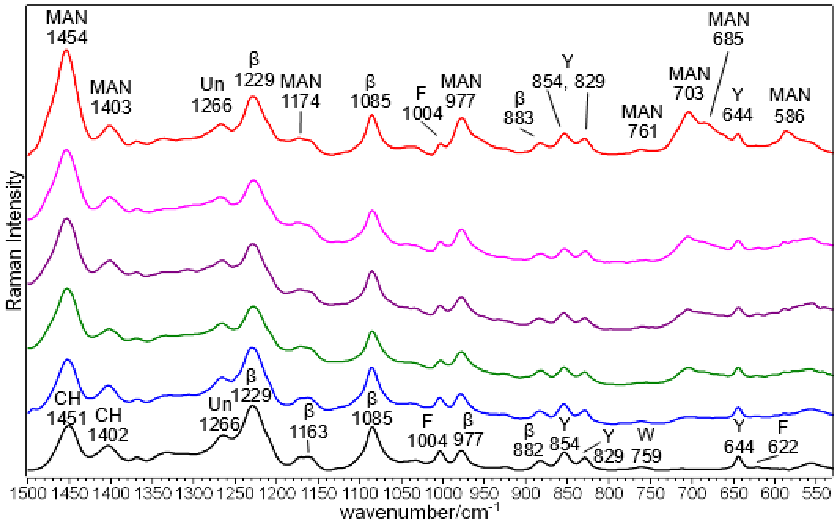

(**c**)

Figure S1. Raman spectra of *B. mori* silk fibroin fibers before and after grafting with MAN at different weight gains (i.e., 10%, 25%, 32.5%, 40%, and 60%) in three different spectral ranges: (**a**) 3350-2180 cm^{-1}, (**b**) 1730-1530 cm^{-1}, and (**c**) 1500-530 cm^{-1}. The spectra are normalized to the intensity of the Amide I band. The main bands assignable to β-sheet (β) or unordered (Un) conformation as well as to tyrosine (Y), phenylalanine (F), tryptophan (W), and the grafting agent (MAN) are indicated.

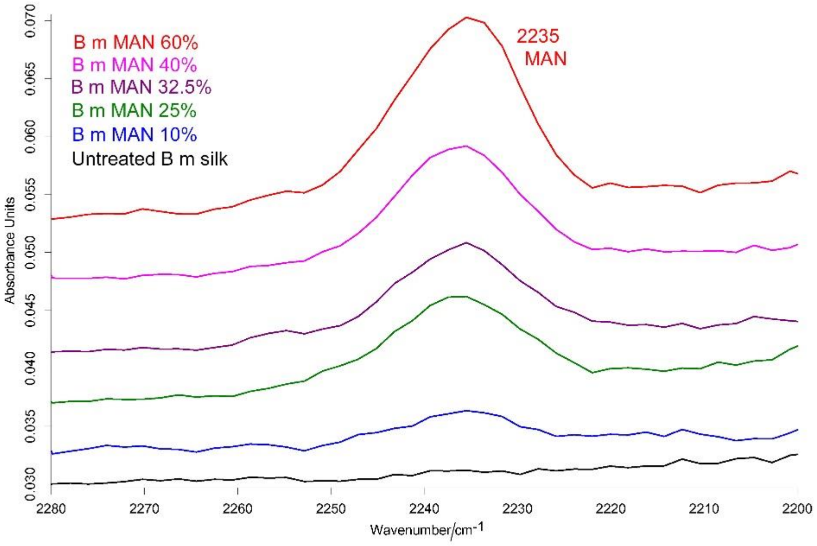

(**a**)

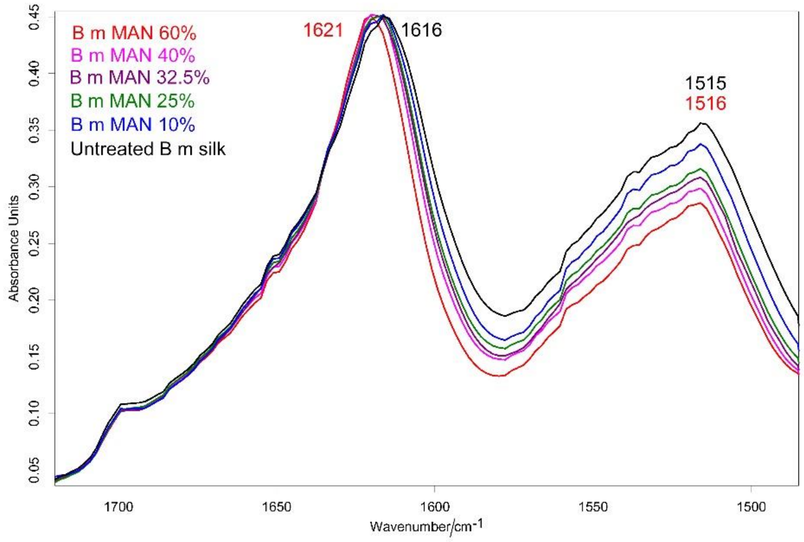

(**b**)

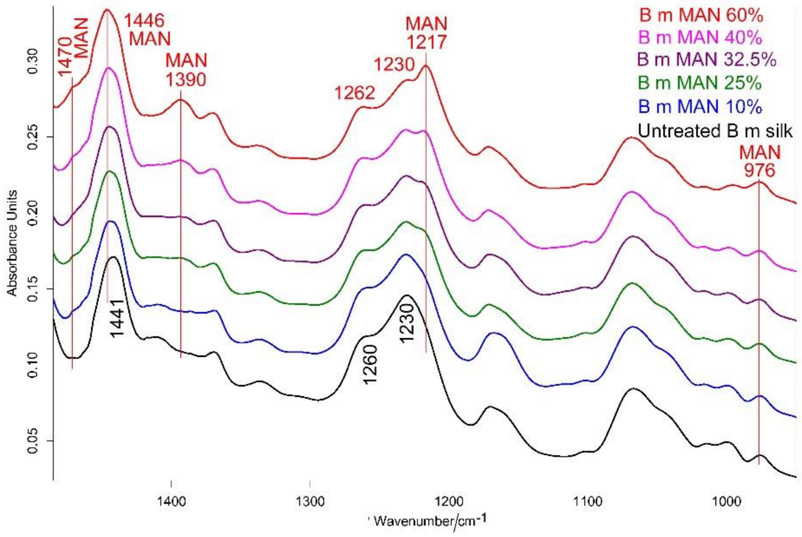

(**c**)

Figure S2. IR spectra recorded in the parallel orientation on *B. mori* silk fibroin fibers before and after grafting with MAN at different weight gains (i.e., 10%, 25%, 32.5%, 40%, and 60%) in three different spectral ranges: (**a**) 2280-2200 cm^{-1}, (**b**) 1720-1485 cm^{-1}, and (**c**) 1485-950 cm^{-1}. The spectra are normalized to the intensity of the Amide I band. The main bands that underwent changes upon MAN grafting are indicated.

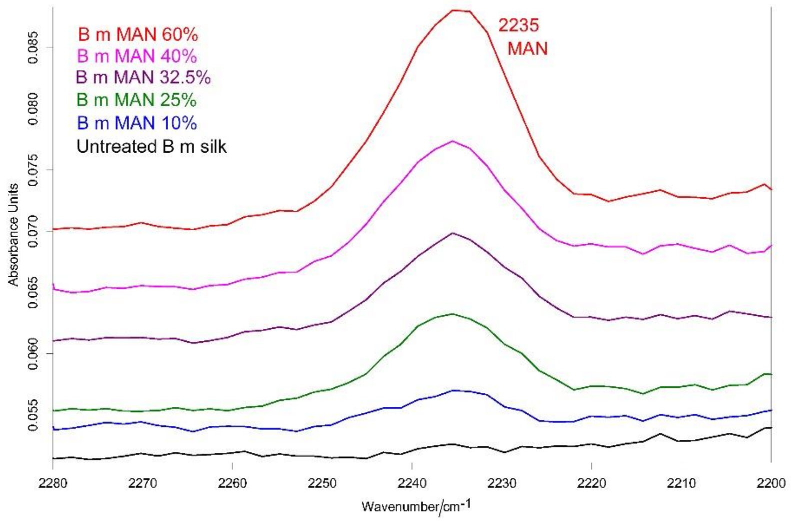

(**a**)

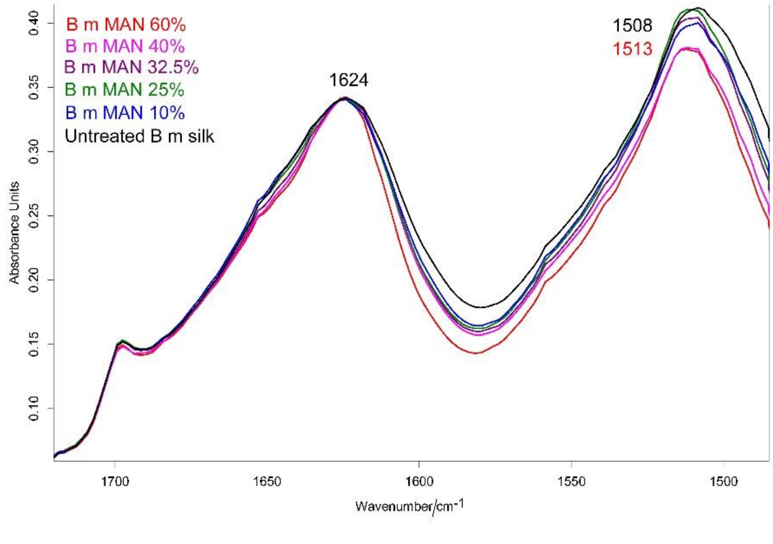

(**b**)

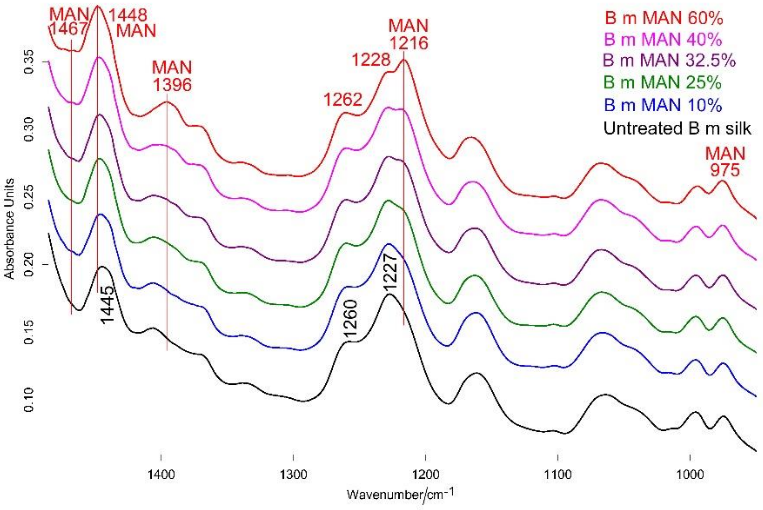

(**c**)

Figure S3. IR spectra recorded in the perpendicular orientation on *B. mori* silk fibroin fibers before and after grafting with MAN at different weight gains (i.e., 10%, 25%, 32.5%, 40%, and 60%) in three different spectral ranges: (**a**) 2280-2200 cm^{-1}, (**b**) 1720-1485 cm^{-1}, and (**c**) 1485-950 cm^{-1}. The spectra are normalized to the intensity of the Amide I band. The main bands that underwent changes upon MAN grafting are indicated.

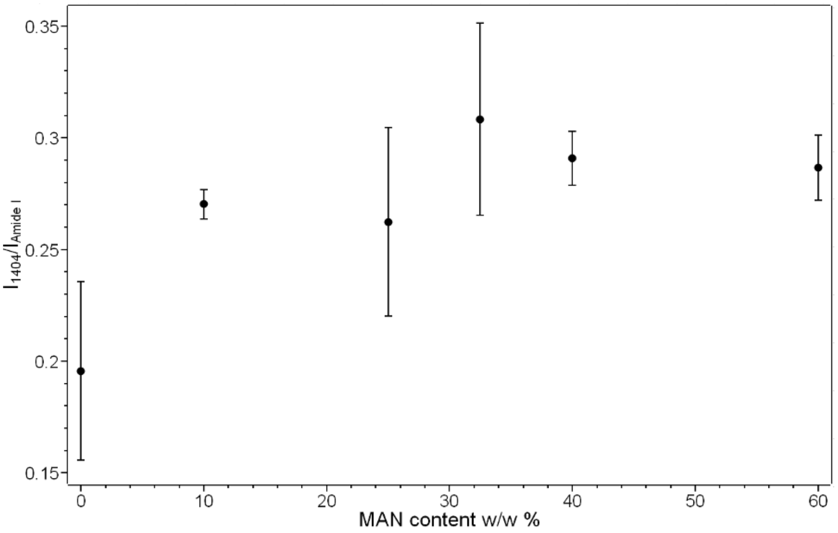

Figure S4. Trend of the $I_{1404}/I_{Amide\ I}$ Raman intensity ratio as a function of the weight gain (i.e., MAN content w/w %) for the silk fibroin fibers grafted with MAN.

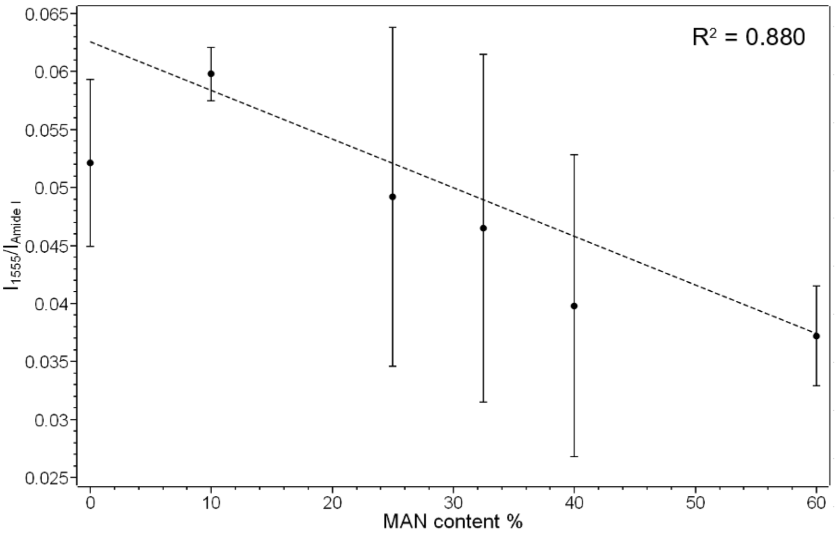

Figure S5. Trend of the $I_{1555}/I_{Amide\ I}$ Raman intensity ratio as a function of the weight gain (i.e., MAN content w/w %) for the silk fibroin fibers grafted with MAN.

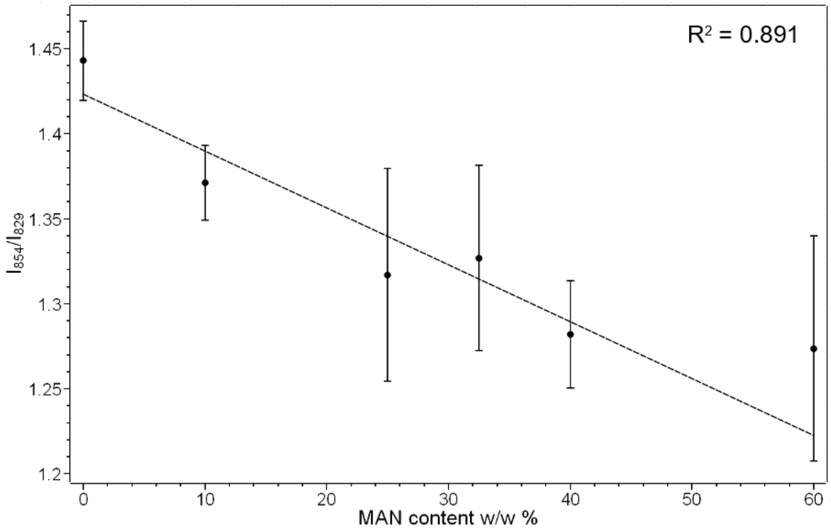

Figure S6. Trend of the I_{854}/I_{829} Raman intensity ratio as a function of the weight gain (i.e., MAN content w/w %) for the silk fibroin fibers grafted with MAN.

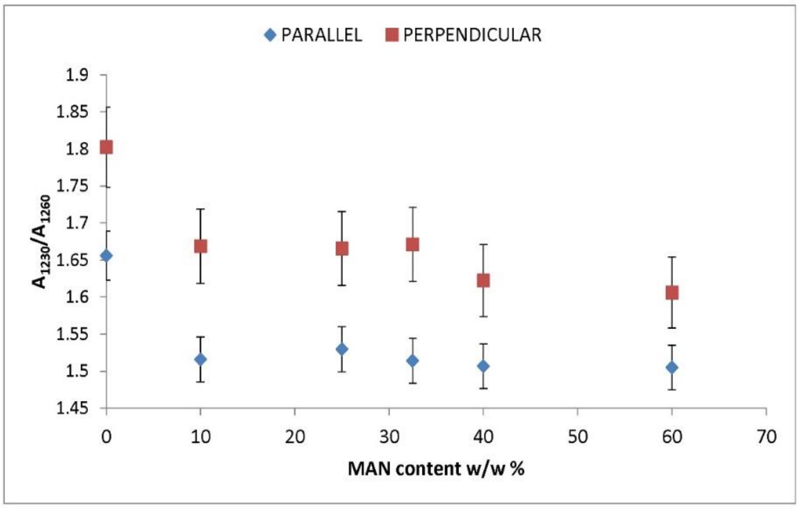

Figure S7. Trend of the A_{1230}/A_{1260} absorbance ratio as a function of the weight gain (i.e., MAN content w/w %).

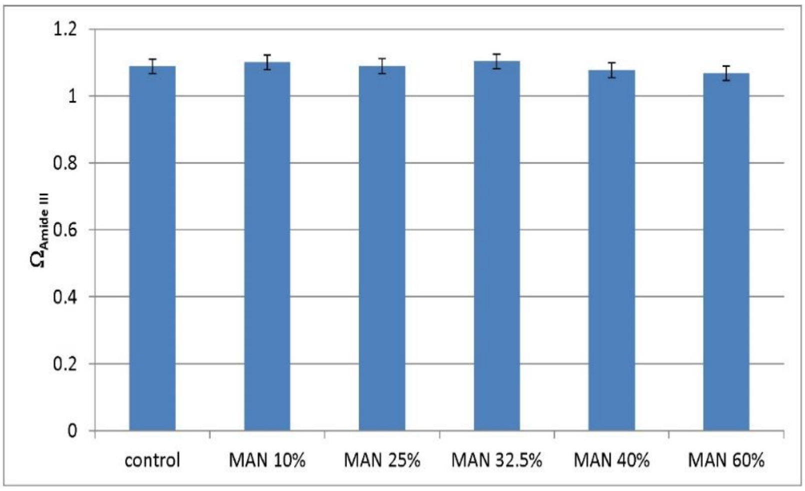

Figure S8. Values of the $\Omega_{\text{Amide III}}$ marker as obtained from the IR spectra recorded in the parallel and perpendicular orientations on the samples under study (control = untreated fibers, and MAN grafted fibers containing different amounts of MAN).

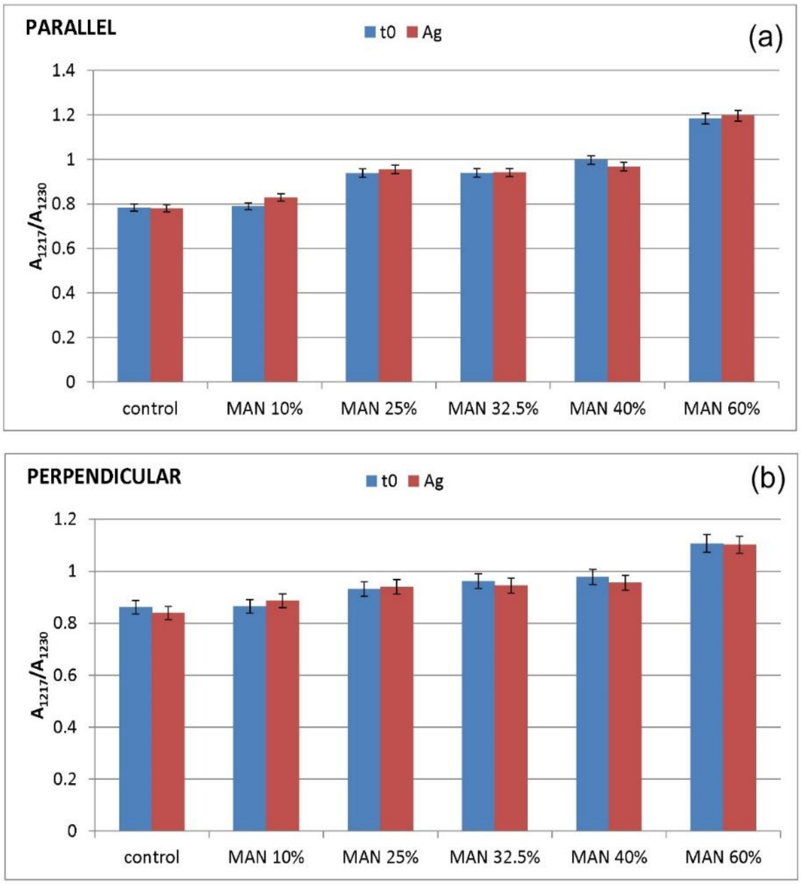

Figure S9. Values of the A_{1217}/A_{1230} absorbance ratio as obtained from the IR spectra recorded in the parallel (**a**) and perpendicular (**b**) orientations on the samples under study (control = untreated fibers, and MAN grafted fibers containing different amounts of MAN) before (t0) and after ageing of the fibers in the Ag^+ containing solution for 48h.

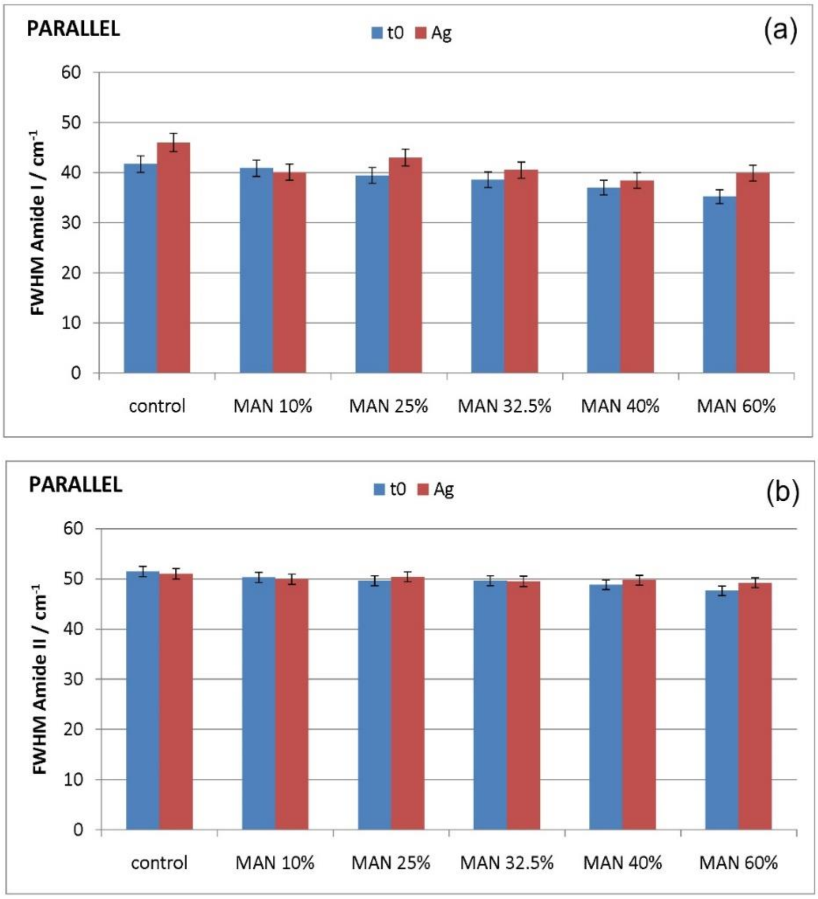

Figure S10. Values of the FWHM of Amide I (**a**) and Amide II (**b**) as obtained from the IR spectra recorded in the parallel orientation on the samples under study (control = untreated fibers, and MAN grafted fibers containing different amounts of MAN) before (t0) and after ageing of the fibers in the Ag^{+} containing solution for 48h.

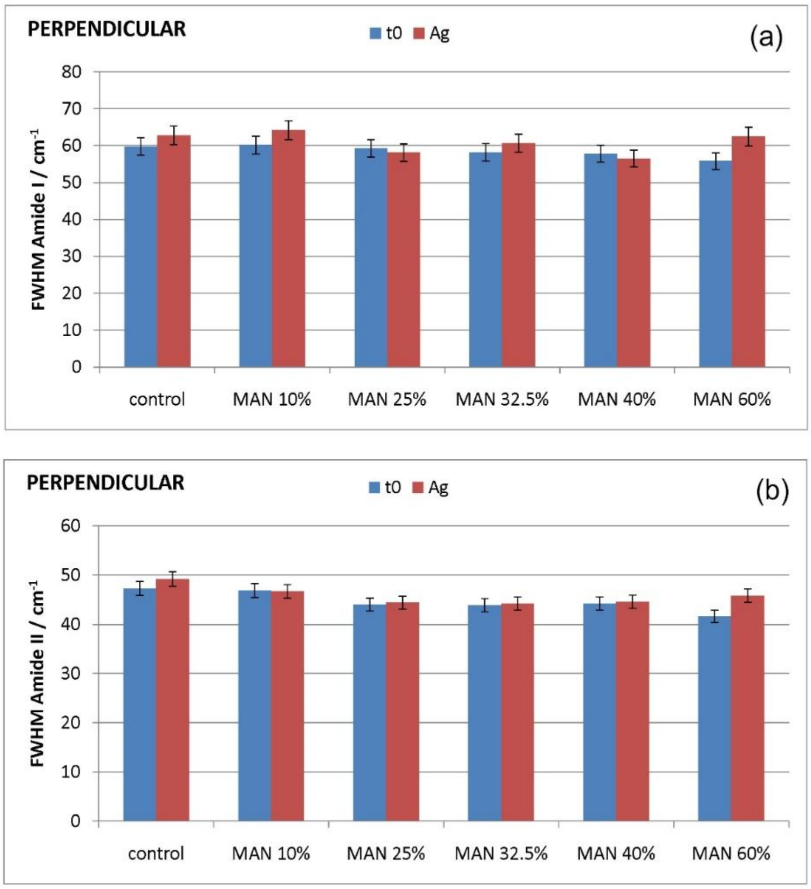

Figure S11. Values of the FWHM of Amide I (**a**) and Amide II (**b**) as obtained from the IR spectra recorded in the perpendicular orientation on the samples under study (control = untreated fibers, and MAN grafted fibers containing different amounts of MAN) before (t0) and after ageing of the fibers in the Ag^{+} containing solution for 48h.

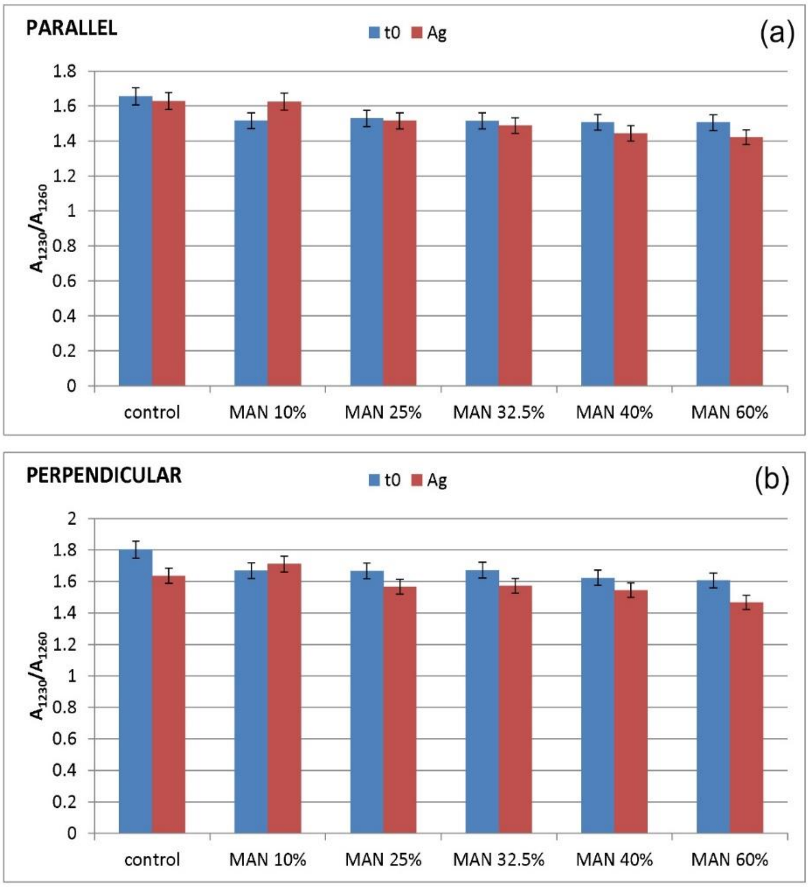

Figure S12. Values of the A_{1230}/A_{1260} absorbance ratio as obtained from the IR spectra recorded in the parallel (**a**) and perpendicular (**b**) orientations on the samples under study (control = untreated fibers, and MAN grafted fibers containing different amounts of MAN) before (t0) and after ageing of the fibers in the Ag^{+} containing solution for 48h.

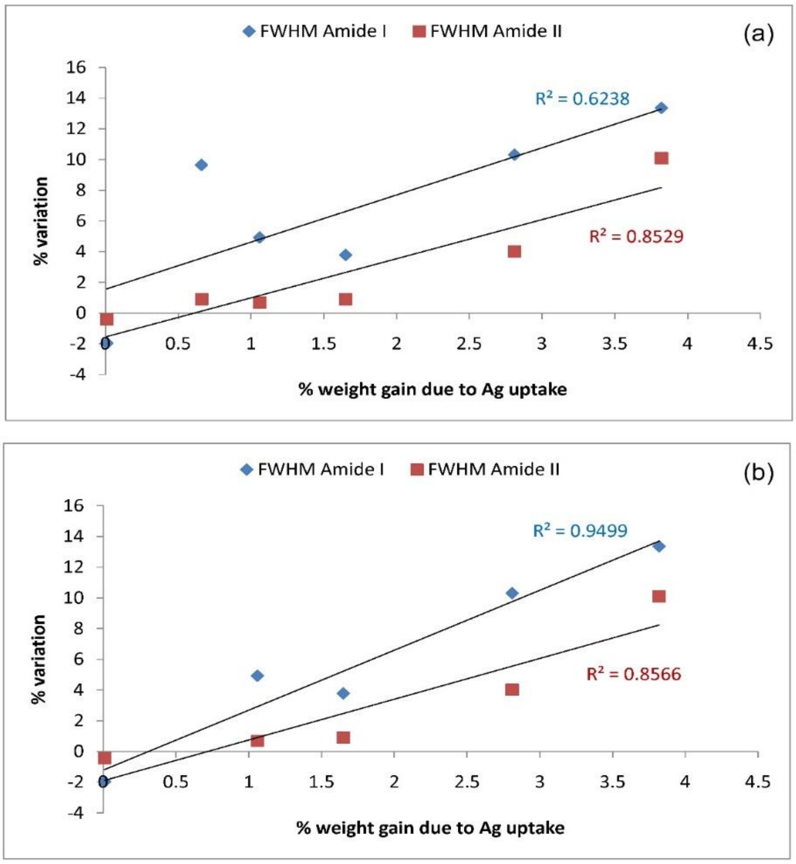

Figure S13. Trend of the % variation of the FWHM of Amide I (in the parallel spectra) and Amide II (in the perpendicular spectra) upon ageing in the Ag^+ solution for 48h, as a function of the % weight gain due to silver uptake. Graph (**a**) reports data from all the samples, while (**b**) reports trends calculated excluding the 25% MAN-grafted sample (outlier).

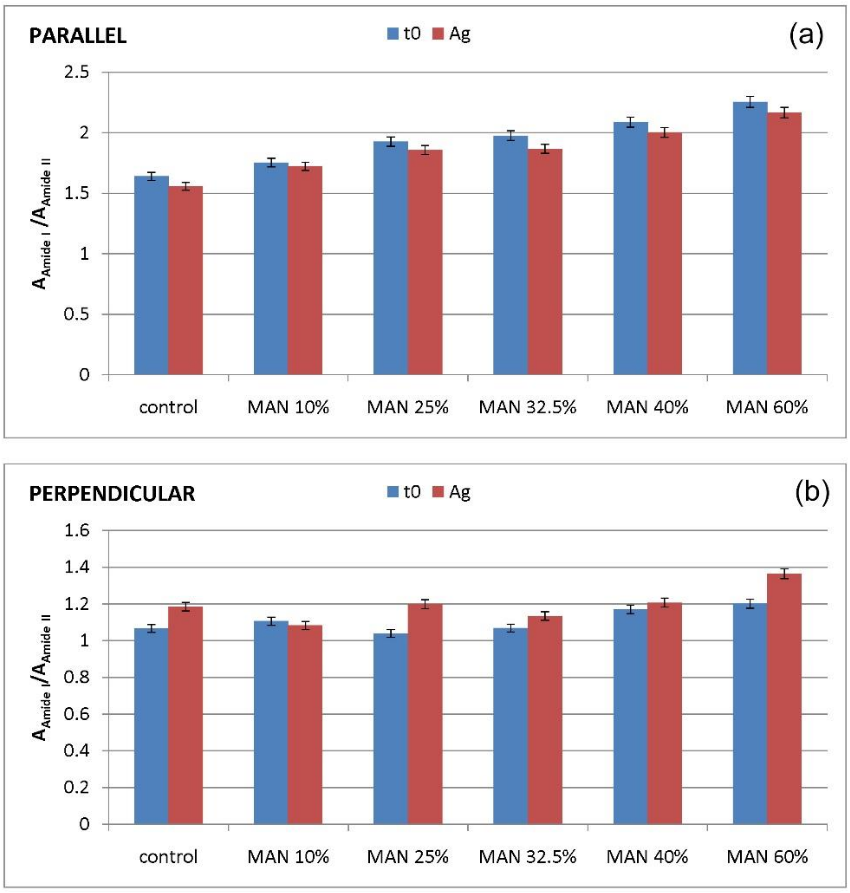

Figure S14. Values of the $A_{Amide\ I}/A_{Amide\ II}$ absorbance ratio as obtained from the IR spectra recorded in the parallel (**a**) and perpendicular (**b**) orientations on the samples under study (control = untreated fibers, and MAN grafted fibers containing different amounts of MAN) before (t0) and after ageing of the fibers in the Ag^{+}-containing solution for 48h.

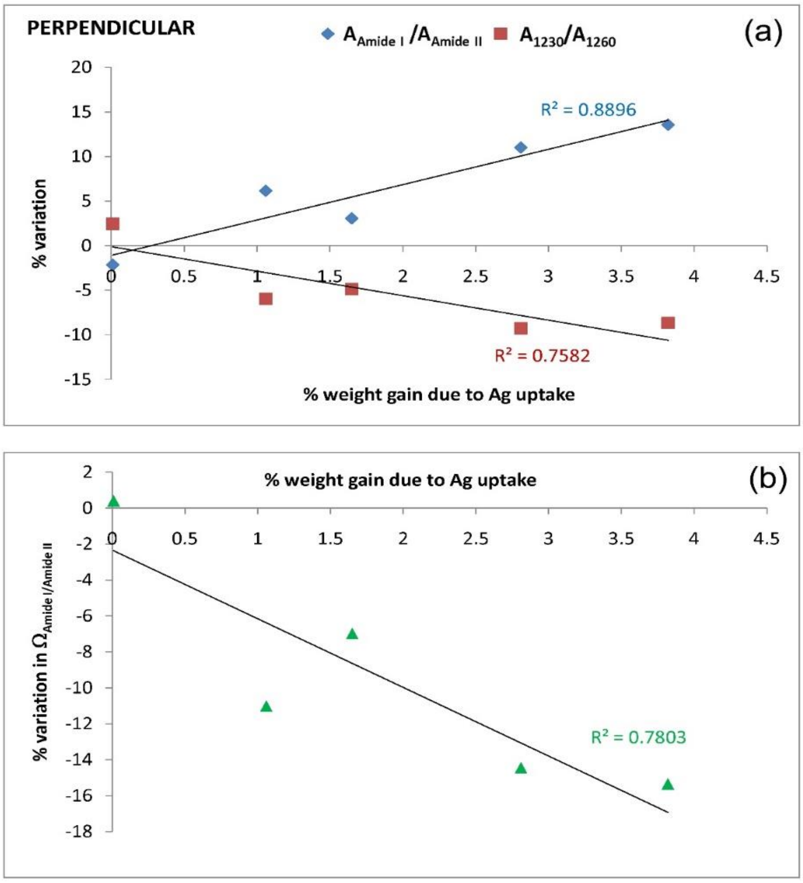

Figure S15. (**a**) Trend of the % variation of the $A_{Amide\ I}/A_{Amide\ II}$ and A_{1230}/A_{1260} absorbance ratios (in the perpendicular spectra) and (**b**) $\Omega_{Amide\ I/Amide\ II}$ upon ageing in the Ag^+ solution for 48h, as a function of the % weight gain due to silver uptake.

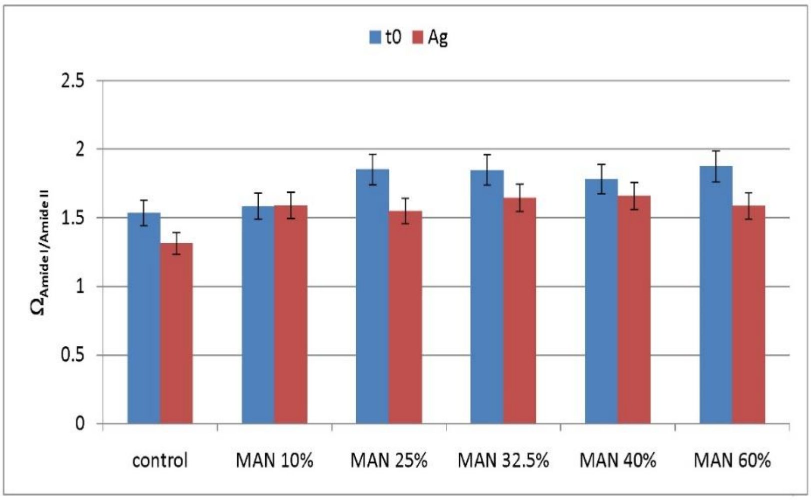

Figure S16. Values of $\Omega_{Amide\ I/Amide\ II}$ as obtained from the IR spectra recorded in the parallel and perpendicular orientations on the samples under study (control = untreated fibers, and MAN grafted fibers containing different amounts of MAN) before (t0) and after ageing of the fibers in the Ag^+ containing solution for 48h.

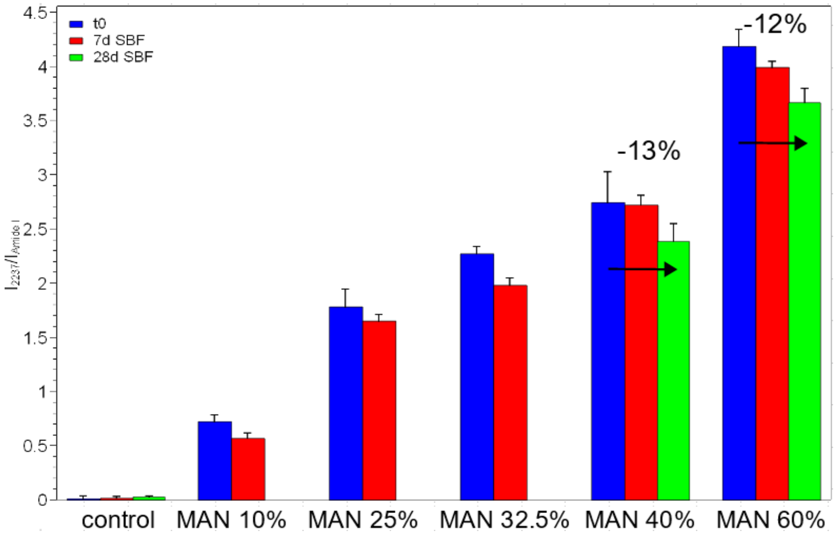

Figure S17. Trend of the $I_{2237}/I_{Amide\ I}$ Raman intensity ratio before and after 7 and 28 days of immersion in the SBF solution.

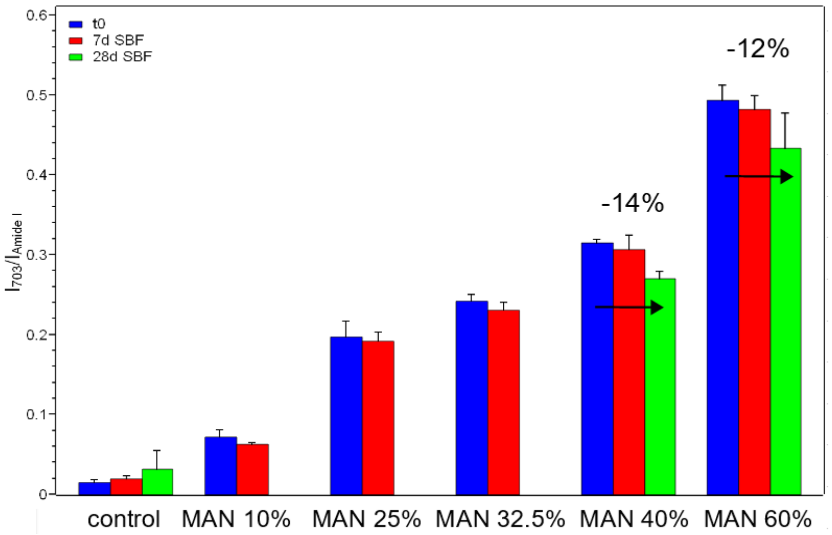

Figure S18. Trend of the $I_{703}/I_{Amide\ I}$ Raman intensity ratio before and after 7 and 28 days of immersion in the SBF solution.

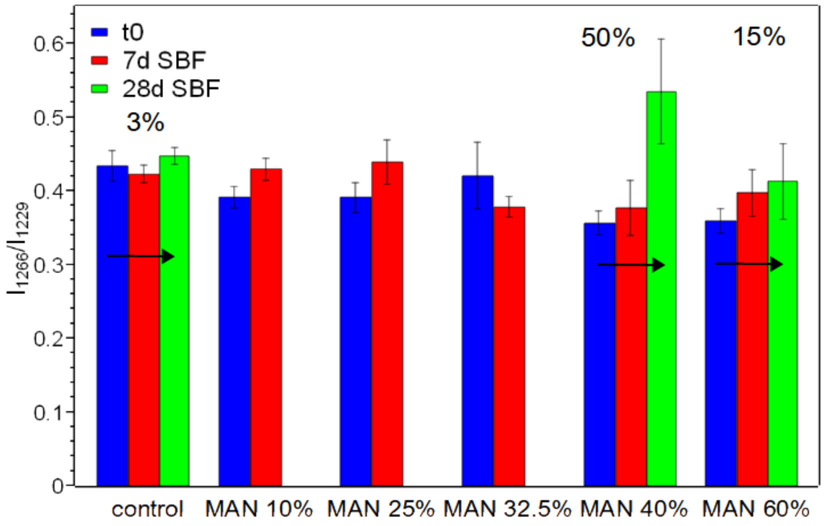

Figure S19. Trend of the I_{1266}/I_{1229} Raman intensity ratio before and after 7 and 28 days of immersion in the SBF solution.

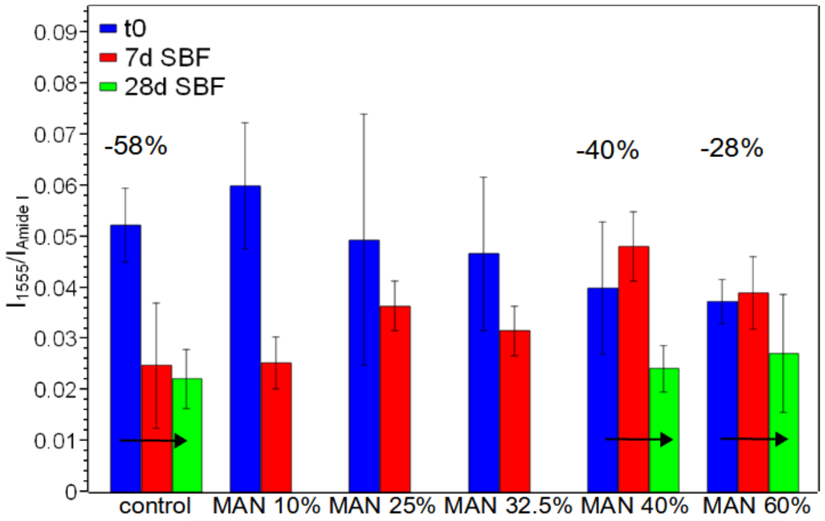

Figure S20. Trend of the $I_{1555}/I_{Amide\ I}$ Raman intensity ratio before and after 7 and 28 days of immersion in the SBF solution.

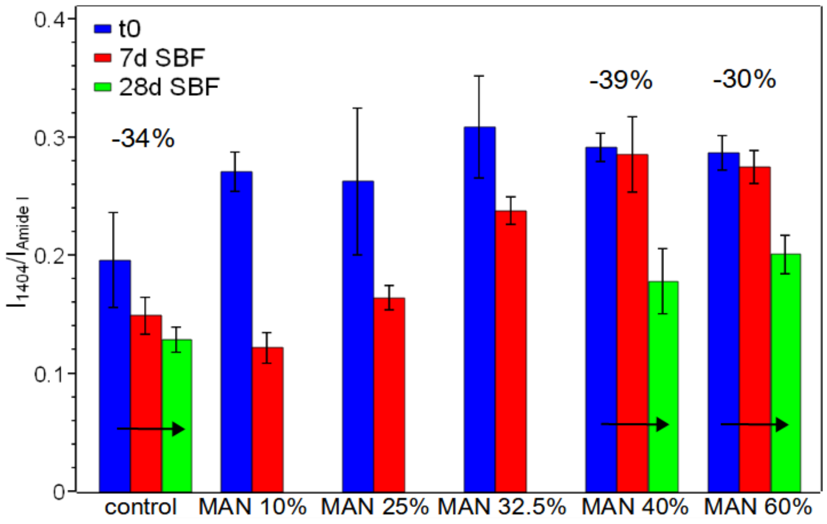

Figure S21. Trend of the $I_{1404}/I_{Amide\ I}$ Raman intensity ratio before and after 7 and 28 days of immersion in the SBF solution.

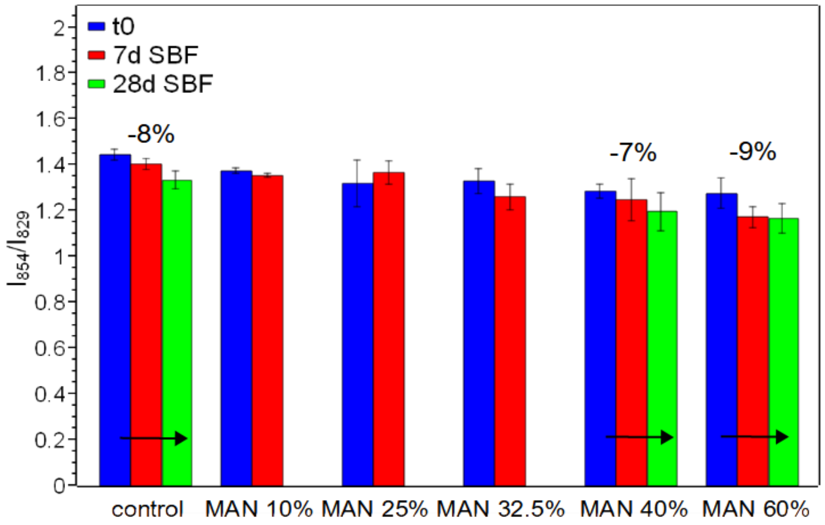

Figure S22. Trend of the I_{854}/I_{829} Raman intensity ratio before and after 7 and 28 days of immersion in the SBF solution.

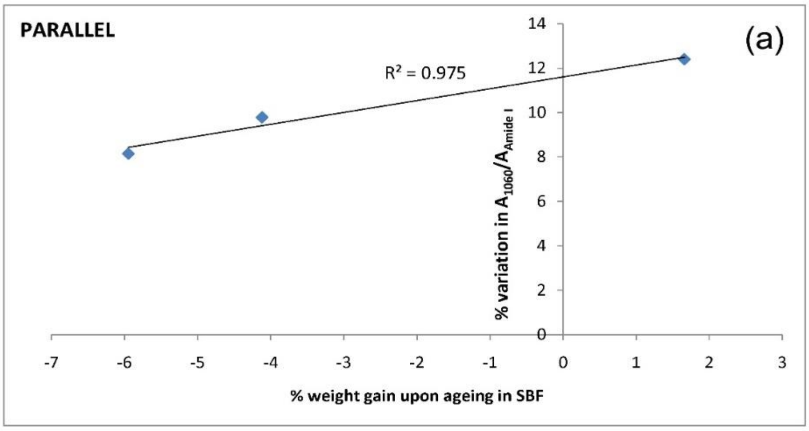

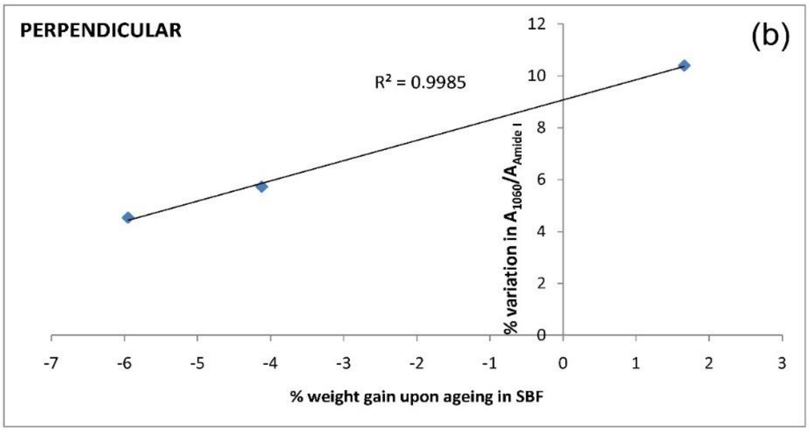

Figure S23. Trend of the % variation of the $A_{1060}/A_{Amide\ I}$ absorbance ratio ((a) parallel spectra; (b) perpendicular spectra) upon ageing in SBF for 28 days, as a function of the % weight gain.

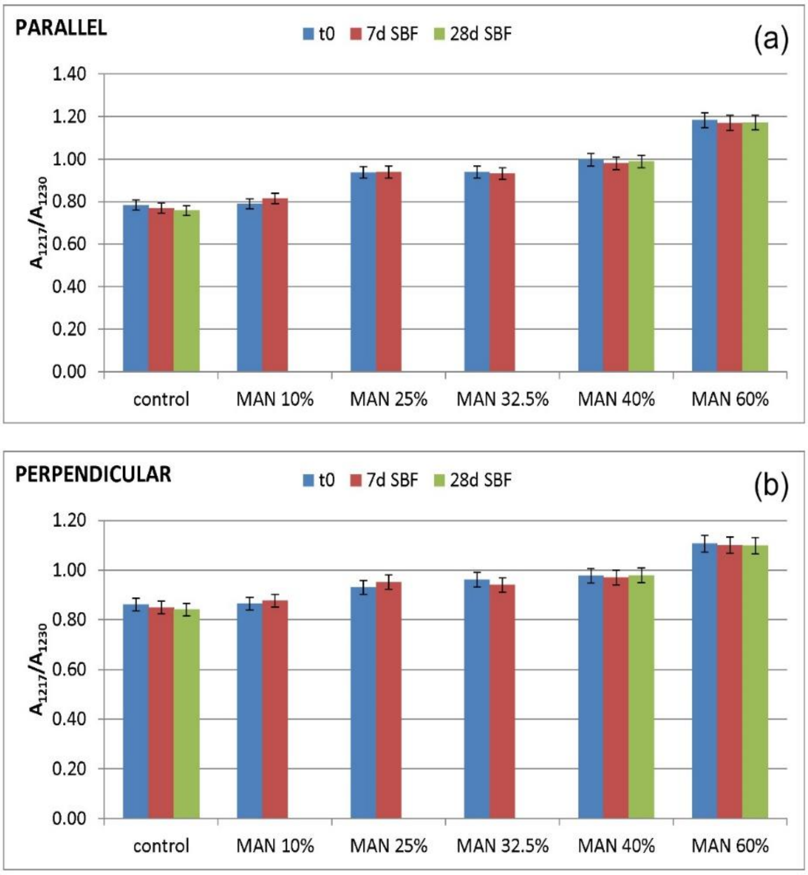

Figure S24. Values of the A_{1217}/A_{1230} absorbance ratio as obtained from the IR spectra recorded in the parallel (**a**) and perpendicular (**b**) orientations on the samples under study (control = untreated fibers, and MAN grafted fibers containing different amounts of MAN) before (t0) and after ageing of the fibers in SBF.

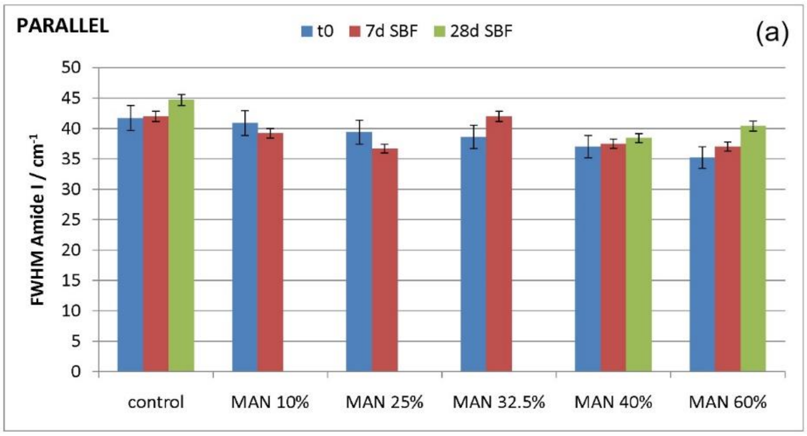

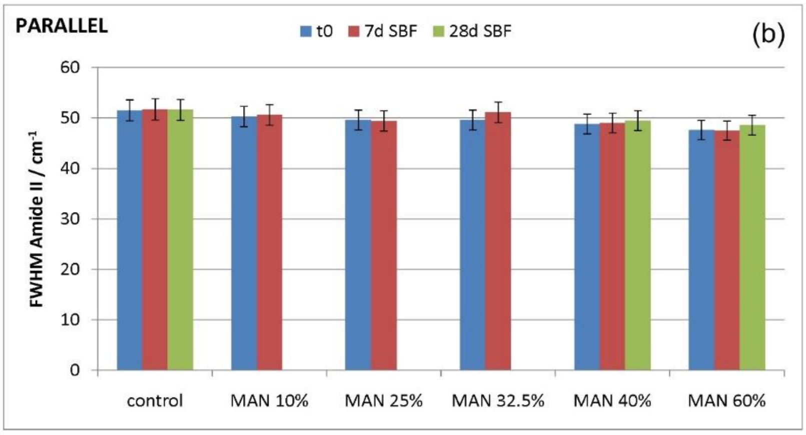

Figure S25. Values of the FWHM of Amide I (**a**) and Amide II (**b**) bands as obtained from the IR spectra recorded in the parallel orientation on the samples under study (control = untreated fibers, and MAN grafted fibers containing different amounts of MAN) before (t0) and after ageing of the fibers in SBF.

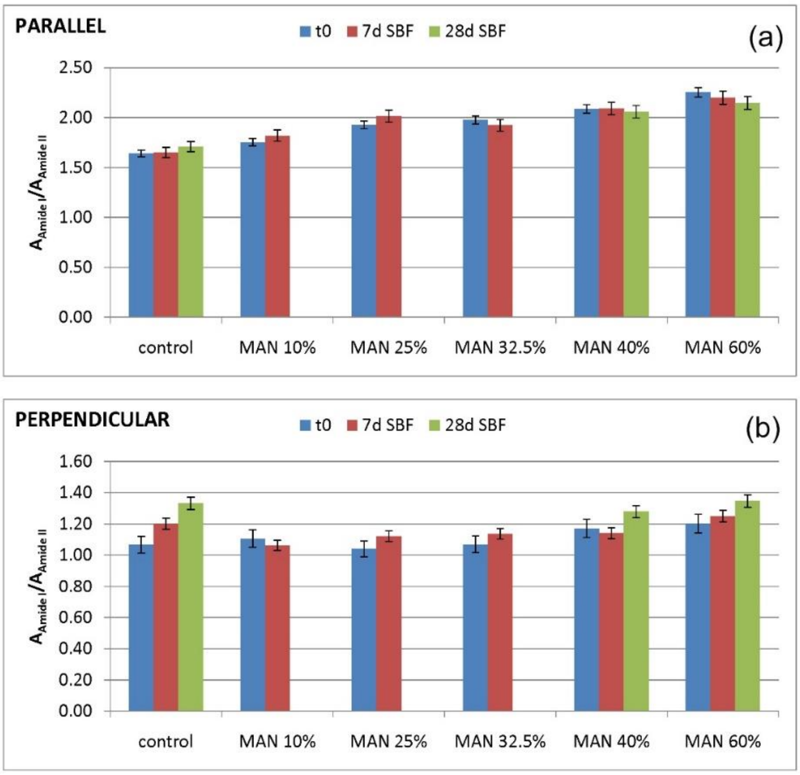

Figure S26. Values of the $A_{\text{Amide I}}/A_{\text{Amide II}}$ absorbance ratio as obtained from the IR spectra recorded in the parallel (**a**) and perpendicular (**b**) orientations on the samples under study (control = untreated fibers, and MAN grafted fibers containing different amounts of MAN) before (t0) and after ageing of the fibers in SBF.

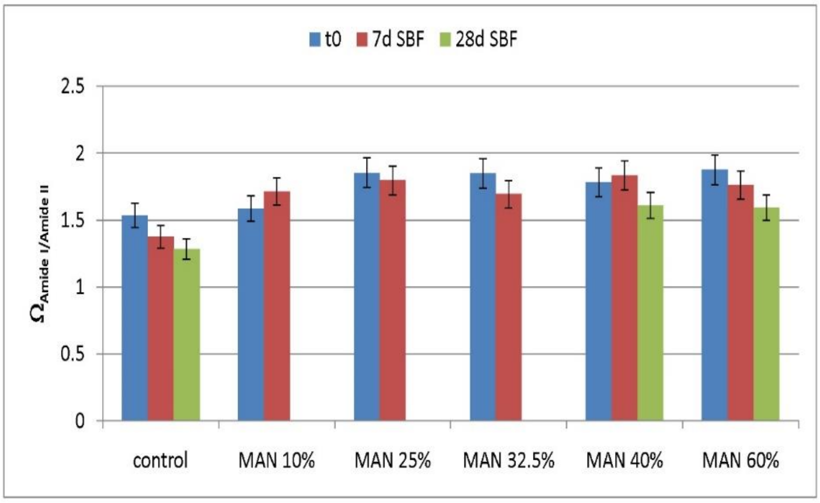

Figure S27. Values of the $\Omega_{\text{Amide I/Amide II}}$ marker as obtained from the IR spectra recorded in the parallel and perpendicular orientations on the samples under study (control = untreated fibers, and MAN grafted fibers containing different amounts of MAN) before (t0) and after ageing of the fibers in SBF.

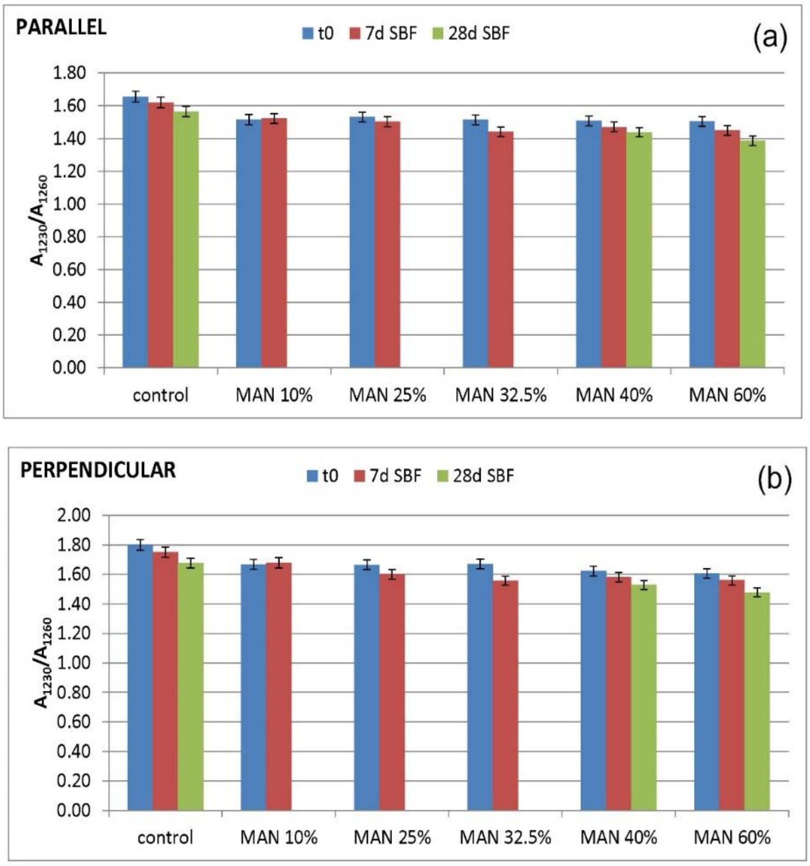

Figure S28. Values of the A1230/A1260 absorbance ratio as obtained from the IR spectra recorded in the parallel (**a**) and perpendicular (**b**) orientations on the samples under study (control = untreated fibers, and MAN grafted fibers containing different amounts of MAN) before (t0) and after ageing of the fibers in SBF.

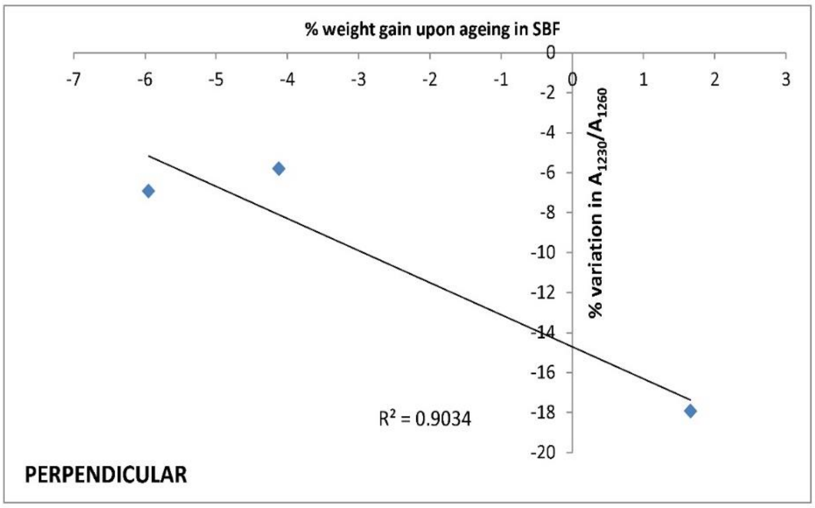

Figure S29. Trend of the % variation of the A1230/A1260 absorbance ratio upon ageing in SBF for 28 days as a function of the % weight gain (perpendicular spectra).
